# Card-placing test in amnestic mild cognitive impairment and its neural correlates

**DOI:** 10.1186/1471-2377-14-123

**Published:** 2014-06-06

**Authors:** Seong-Joon Lee, Young-Sil An, Tae Sung Lim, So Young Moon

**Affiliations:** 1 Department of Neurology, School of Medicine, Ajou University, 5 San, Woncheon-dong, Yongtong-gu, Suwon-si, Kyunggi-do 442-749, Republic of Korea; 2 Nuclear Medicine, Ajou University School of Medicine, Suwon, Republic of Korea

**Keywords:** Mild cognitive impairment, Heading disorientation, Posterior cingulate gyrus, Card placing test

## Abstract

**Backgroud:**

We investigated anatomical correlates of the card-placing test (CPT) in patients with amnestic mild cognitive impairment (aMCI).

**Methods:**

Fifteen aMCI patients underwent part A and part B of the CPT and FDG-PET. The CPT scores and MMSE scores of 29 cognitively normal people were used for comparison. Statistical parametric mapping (SPM) correlation analysis was used to extract the regions whose changes in regional cerebral metabolism correlated significantly with part A and B of the CPT with adjustment of age, education and sex of patients.

**Results:**

The aMCI patients had significantly lower MMSE scores (26.0 ± 2.0 vs. 28.2 ± 1.4, p < 0.001), CPT A (25.5 ± 3.5 vs. 27.7 ± 2.7, p = 0.026) and CPT B scores (16.3 ± 4.4 vs. 19.7 ± 3.7, p = 0.011) compared to the normal population. The test scores of part B of the CPT correlated well with hypometabolism of the posterior cingulate gyrus and precuneus.

**Conclusions:**

This study suggests that the CPT B may reflect the functional status of the posterior cingulate gyrus in patients with aMCI.

## Background

'Topographically disoriented' patients lose their ability to find their way within large-scale, locomotor environments. Heading disorientation (HD) is a type of pure topographical disorientation [1]. Patients with HD can represent the relative locations of objects and they are able to easily recognize landmarks, but suffer from topographical impairment not only in a novel but also in a familiar environment. It is implied that the primary deficit in HD is an inability to derive directional information from landmarks to reach a destination [1-3]. In other words, one that can be selectively damaged in HD is exocentric spatial representations, in which spatial relations between objects within the environment, including the observer are emphasized [4]. Although the number of reported cases has been limited so far, the lesion site responsible for HD is presumed to be the posterior cingulate gyrus, which is supposed to be the place where information necessary for navigation converges [1]. However, there had not been a specific test for HD until the card-placing test (CPT) was introduced [3].

The CPT examines the ability to represent spatial locations of objects placed on the floor around a subject. The CPT consists of two parts – part A and B. Part A of the test assesses the ability of a subject to retain information on spatial locations of cards placed on the floor around her/him. Part B examines the subject's ability to integrate information on the spatial locations of similarly arranged cards and that on changes of the body directions. Hashimoto et al. [3] reported that three patients with HD after damages in the right retrosplenial cortex showed good performance for part A but very poor one for part B. In the CPT, patients with HD were defective in the processing of directional signals of the self, or unable to integrate information on the registered external spatial locations of objects with that on their body direction. In addition, it was suggested that the CPT could be used to evaluate the functional state of right retrosplenial cortex. The retrosplenial region is a brain area and part of the cingulate cortex. It is defined by Brodmann area 26, Brodmann area 29 and Brodmann area 30.

The posterior cingulate gyrus, is of much interest because metabolic reduction in that area is seen in very early Alzheimer’s disease (AD) and its presence has also been proven to predict progression to AD in mild cognitive impairment (MCI) [5-7]. Considering that performance in the part B of the CPT was impaired in patients with right retrosplenial lesions, the CPT presumes to be the tests to evaluate the functional status of the posterior cingulate gyrus in patients with MCI. However, the CPT has not been used in patients with MCI. In addition, neural substrates of the test have not been replicated in other studies yet. In our study, using statistical parametric mapping (SPM) analysis we aimed to investigate anatomical correlates of the CPT in fluoro-deoxy-glucose positron emission tomography (FDG-PET) studies of patients with amnestic MCI (aMCI).

## Methods

### Participants

Patients were 15 individuals (eight men and seven women) with aMCI. Diagnostic evaluation of patients included a complete medical history, physical and neurologic examinations, neuropsychological tests, magnetic resonance image (MRI) scans, and blood tests which included complete blood counts, blood chemistry profiles, vitamin B_12_/folate levels, syphilis serology, and thyroid function tests. The aMCI patients were diagnosed according to the criteria proposed by Petersen et al. [8]. We excluded patients with a history of significant hearing or visual impairment that rendered interview participation difficult, as well as those with a history of neurological disorders (e.g., active epilepsy), psychiatric illnesses (e.g., schizophrenia, mental retardation, anxiety disorders, major depression, and mania), those taking psychotropic medications, and those with a history of significant alcohol and/or other substance abuse. In addition, twenty nine healthy volunteers (12 men and 17 women) participated. All subjects had corrected visual acuity of 20/40 or better, and no other known neurological or ophthalmological conditions. Controls were free of cognitive problems, which was confirmed by no cognitive complaints as well as by not scoring less than the 16^th^ percentile of the norms for the age-, sex-, and education-matched normal subjects on the Korean version of the Mini-Mental State Examination (MMSE) [9]. We obtained informed consent from all the patients and controls, and the study was approved by the Institutional Review Board of Ajou University Hospital. If participants had impaired decisional capacity, caregivers provided consent and patients provided assent.

### Neuropsychological tests

All patients underwent neuropsychological tests using a standardized neuropsychological battery, called the Seoul Neuropsychological Screening Battery (SNSB) [10]. This battery contained tests for attention, language, praxis, the four symptoms of Gerstmann syndrome, visuoconstructive function, verbal and visual memory, and frontal/executive function. Memory function was evaluated by the delayed recall on the Seoul Verbal Learning Test (SVLT) or Rey-Osterrieth Complex Figure Test (RCFT). Language was assessed by the Korean version of the Boston Naming Test (K-BNT). Visuospatial function was evaluated by the copying score of the RCFT. Finally, frontal/executive tests were classified into three groups: motor executive function (contrasting program, go/no-go, fist-edge-palm, alternating hand movement, alternative square and triangle, and Luria loop), Controlled Oral Word Association Test (COWAT), and Stroop Test. Impaired frontal/executive function was operationally defined as impairment in at least two of the three groups. Among neuropsychological tests, scorable tests included the digit span test (forward and backward), the K-BNT, the RCFT (copying, immediate and 20-minute delayed recall, and recognition), the SVLT (three free recall trials of 12 words, and a 20-minute delayed recall trial of the same 12 items, and a recognition test), the phonemic and semantic COWAT, and the Stroop Test (word and color reading of 112 items for two minutes). The scores on cognitive tests were classified as abnormal when they were below the 16^th^ percentile of the norms for the age-, sex-, and education-matched normal subjects. On the basis of the results of the SNSB, aMCI patients were diagnosed with amnestic MCI-single domain or amnestic MCI-multiple domains.

### CPT

Participants underwent the CPT as previously described [3] in a featureless room. In part A of the CPT, a subject stands in the center square of nine squares arranged in three rows of three. The subject was instructed to remember the spatial locations of three different cards randomly placed in one of the eight squares. After 10 s, the cards were taken away and the subject is to restore them to their original positions. In part B, immediately after the cards had been removed, the subject was rotated to the right or to the left by 90 or 180°, and then asked to replace the cards. For both part A and part B of the CPT, the subject underwent 10 consecutive trials, and scored 1 point if the location of a card that the subject replaced was correct. The full score of each of part A and part B of the CPT was 30 points.

### FDG PET studies

Fifteen patients with aMCI received FDG-PET. PET/CT data were acquired on a Discovery ST scanner (General Electric Medical Systems, USA). After fasting for at least 4 h, patients received 300 MBq of FDG intravenously. We checked serum glucose-levels in all subjects prior to FDG injection, and the subjects whose glucose-level exceeds 150 mg/dl were excluded. All subjects were instructed to rest comfortably for 30 min with their eyes closed and ears unplugged and then image acquisition was started. To reduce head movement during scanning, the patients were positioned and maintained using an individually molded head holder. They first had a CT scan (tube-rotation time of 1 s per revolution, 120 kV, 70 mA, 5.0 mm per rotation and an acquisition time of 11.8 s for a scan length of 150.42 mm). Subsequently, one frame (8 min per frame) of emission PET data was acquired in a three-dimensional mode. PET images were reconstructed by iterative reconstruction (ordered subsets expectation maximization, with one iteration and 32 subsets), using the CT images for attenuation correction. Also, the random correction by singles and model-based scatter correction were applied.

### FDG PET data analysis

FDG-PET images were spatially normalized to a standard template provided by SPM2 (Statistical Parametric Mapping 2, Institute of Neurology, University of London, UK) on MATLAB (version 7.1; Mathworks, Natick, MA). A local optimization of the 12 parameters of an affine transformation was applied to spatial normalization. These images were then smoothed with a Gaussian kernel (full-width at half-maximum = 16 mm) to minimize noise and improve between-subject spatial alignment. Appropriate voxel-by-voxel statistical tests were used to evaluate differences in glucose metabolism. A correlation analysis was performed to extract regions whose changes in regional cerebral metabolism correlated significantly with part A and part B of the CPT as well as scorable neuropsychological tests and MMSE, controlling for age, education and sex. Anatomical labeling of significant clusters was performed using automated anatomical labeling SPM toolbox, which was based on anatomy provided by the Montreal Neurological Institute.

### Statistical analyses

We used chi square and the student’s t-test to compare demographic data and test scores between patients and normal population. A p-values <0.05 were deemed significant. The statistical analysis was performed using commercially available software (SPSS, version 18.0). In the SPM analyses, regions reaching uncorrected threshold of p < 0.005, were considered to be significant due to the small number of the patient population.

## Results

### aMCI patients versus controls

The demographics of the patients and the normal population are outlined in the Table 1. There was no statistically significant difference in their age, sex and education between aMCI and normal controls (p > 0.05). The aMCI patients had significantly lower MMSE scores (26.0 ± 2.0 vs. 28.2 ± 1.4, p < 0.001), CPT A (25.5 ± 3.5 vs. 27.7 ± 2.7, p = 0.026) and CPT B scores (16.3 ± 4.4 vs. 19.7 ± 3.7, p = 0.011) compared to the normal population. The aMCI patients comprised of 10 (66.7%) single domain aMCI, and five (33.3%) multiple domain aMCI patients. Multiple domain aMCI patients were older (years old, 67.0 ± 8.1 vs 65.1 ± 4.3, p = 0.559), more highly-educated (years, 10.0 ± 3.8 vs 8.8 ± 5.2, p = 0.672), but more impaired in both CPT A (23.6 ± 3.7 vs. 26.4 ± 3.0, p = 0.148) and CPT B (14.4 ± 5.2 vs. 17.3 ± 3.8, p = 0.241) than single domain aMCI patients, although their difference was not statistically significant.

**Table 1:** Demographic findings and test results of normal population and aMCI patients

	**aMCI patients (n = 15)**	**Normal population (n = 29)**	**p-value**
Age, years	65.3 ± 5.5	68.7 ± 5.5	0.066
Sex, male (%)	8 (53.3%)	12 (41.4%)	0.450
Education, years	9.2 ± 4.7	11.7 ± 4.7	0.111
MMSE	26.0 ± 2.0	28.2 ± 1.4	<0.001
Part A, CPT (30)	25.5 ± 3.5	27.7 ± 2.7	0.026
Part B, CPT (30)	16.3 ± 4.4	19.7 ± 3.7	0.011
Attention			
Digit span forward test (9)	5.8 ± 1.4		
Working memory			
Digit span backward test (8)	3.4 ± 0.8		
Language function			
K-BNT (60)	43.2 ± 8.9		
Visuospatial function			
RCFT Copy (36)	31.3 ± 6.2		
Memory function			
SVLT immediate recall (36)	13.4 ± 3.4		
SVLT delayed recall (12)	2.6 ± 1.4		
SVLT recognition (24)	17.2 ± 1.7		
RCFT immediate recall (36)	9.5 ± 5.6		
RCFT delayed recall (36)	8.8 ± 5.1		
RCFT recognition (24)	18.4 ± 2.7		
Frontal function			
Contrasting program (20)	19.2 ± 2.5		
Go-no go (20)	19.6 ± 1.2		
Phonemic COWAT	22.2 ± 6.9		

aMCI, amnestic mild cognitive impairment; Mini-Mental State Examination; CPT, card-placing test; K-BNT, Korean version of Boston Naming Test; RCFT, Rey Complex Figure Test; SVLT, Seoul Verbal Learning Test; COWAT, Controlled Oral Word Association Test.

Data for all variables except for sex: mean ± SD.

The full mark for each test is presented in the brackets.

### SPM correlation analysis

**Figure 1:**
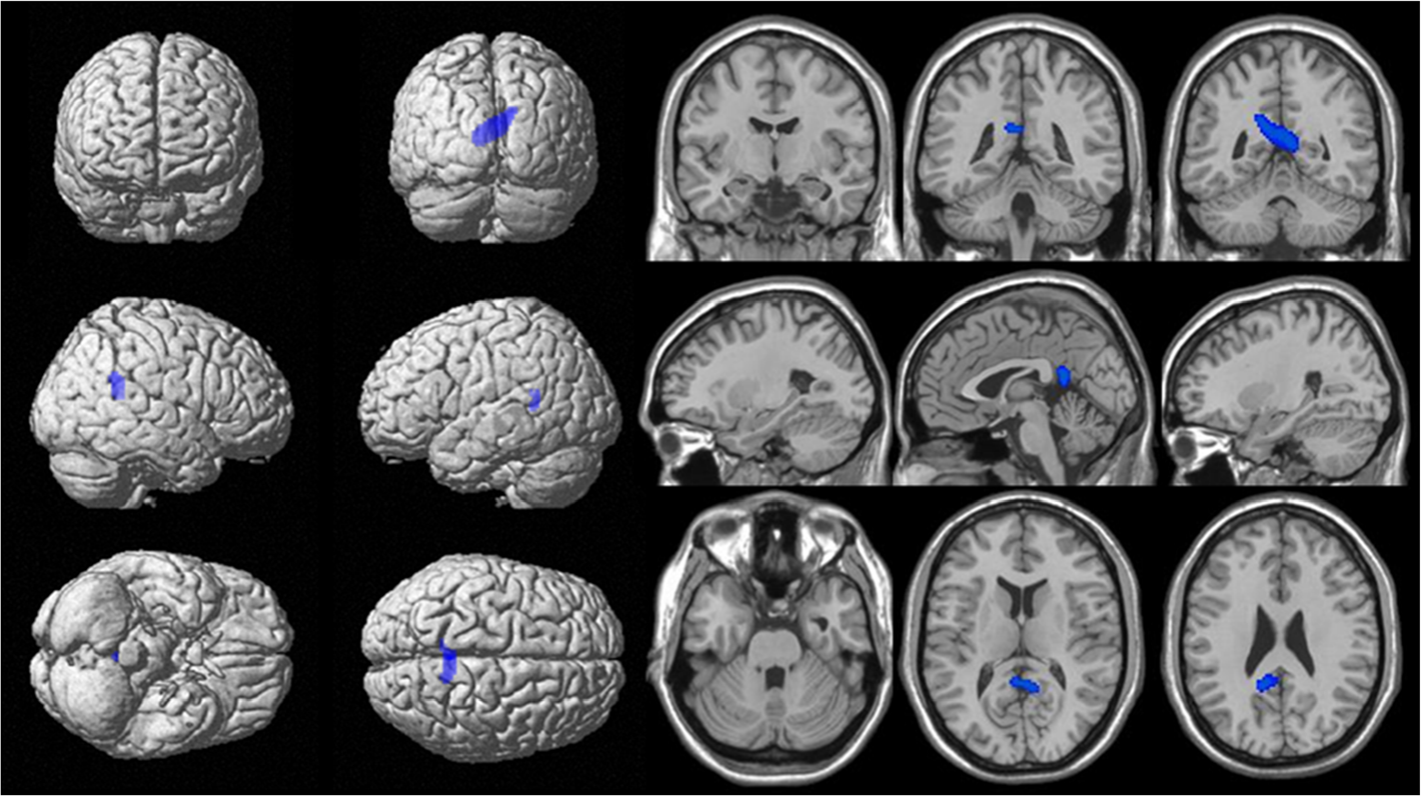
**Spatial parametric maps showing areas of hypometabolism that correlate with decrease in scores of part B of the card-placing Test scores.** Decrease in scores of part B correlated with hypometabolism in bilateral precuneus, middle and posterior cingulate gyri (uncorrected p < 0.005).

Table 2 presents the results of the correlation analysis to evaluate the regions whose changes in regional cerebral metabolism correlated significantly with part B of the CPT. There was no regions whose hypometablism showed correlation with scores of part A. Decrease in scores of part B correlated with hypometabolism in bilateral precuneus, middle and posterior cingulate gyri (Figure 1, uncorrected p < 0.005). Table 3 reveals the results of the correlation analysis to evaluate the regions whose changes in regional cerebral metabolism correlated significantly with scorable neuropsychological tests and MMSE. Any tests except for part B of the CPT did not show correlation with hypometabolism of posterior cingulate gyrus.

**Table 2:** SPM correlation analysis between part B scores of the card-placing test and regional hypometabolism

**Voxel level**	**Voxel level**	**Coordinate**			**Side**	**Area**
**P (uncorrected)**	**P (FDR-corr)**	**X**	**Y**	**Z**		
0.003	0.972	14	-50	28	Right	Precuneus
						Middle cingulate
						Posterior cingulate
0.003	0.972	0	-44	16	Right	Posterior cingulate
						Precuneus
					Left	Posterior cingulate
						Precuneus

FDR-corr, false discovery rate-corrected.

**Table 3:** SPM correlation analysis between neuropsychological tests and regional hypometabolism

**Test**	**Voxel level**	**Voxel level**	**Coordinate**			**Side**	**Area**
	**P (uncorrected)**	**P (FDR-corr)**	**X**	**Y**	**Z**		
Digit span forward	0.001	1.000	46	-46	-40	Right	Cerebellum
	0.001	1.000	-34	6	-50	Left	Middle temporal
Digit span backward	0.001	0.833	24	-34	-28	Right	Cerebellum
	0.002	0.833	18	-40	-18	Right	Cerebellum
K-BNT	<0.001	0.071	2	38	-4	Right	Anterior cingulate
	<0.001	0.074	8	38	16	Right	Anterior cingulate
	<0.001	0.098	-4	38	24	Left	Anterior cingulate
	<0.001	0.071	-38	-6	-50	Left	Inferior temporal
	0.002	0.254	-30	-12	-36	Left	Fusiform
	0.003	0.298	-30	14	-44	Left	Middle temporal
RCFT	0.001	0.158	36	-10	-42	Right	Fusiform
	0.001	0.222	24	-26	-26	Right	Parahippocampal
SVLT immediate recall	0.003	0.999	70	-30	42	Right	Supramarginal
	0.005	0.999	66	-30	50	Right	Supramarginal
	0.001	1.000	30	60	-16	Right	Orbitofrontal
SVLT delayed recall	0.001	1.000	-56	8	38	Left	Precentral
	0.001	1.000	-48	28	30	Left	Inferior frontal
SLVT recognition	<0.001	0.816	24	36	58	Right	Superior frontal
RCFT immediate recall	0.001	0.929	-8	42	2	Left	Anterior cingulate
	0.002	0.929	-8	26	36	Left	Superior frontal
RCFT delayed recall	0.003	0.929	-8	34	26	Left	Anterior cingulate
	0.001	0.880	-8	40	0	Left	Anterior cingulate
	0.003	0.880	8	34	-2	Right	Anterior cingulate
RCFT recognition	<0.001	1.000	-54	22	32	Left	Inferior frontal
	0.002	1.000	6	56	32	Right	Superior frontal
Contrasting program	0.002	1.000	8	42	46	Right	Superior frontal
	<0.001	0.061	-4	30	38	Left	Superior frontal
	<0.001	0.061	-4	40	50	Left	Superior frontal
	<0.001	0.061	-2	24	62	Left	Supplementary motor
	<0.001	0.062	-4	-56	4	Left	Calcarine
	<0.001	0.070	62	18	24	Right	Inferior frontal
	<0.001	0.070	-56	24	22	Left	Inferior frontal
	0.001	0.120	-44	-62	-24	Left	Cerebellum
Go-no go	<0.001	0.438	-46	-14	62	Left	Precentral
	<0.001	0.438	22	42	48	Right	Superior frontal
	0.001	0.438	32	32	54	Right	Middle frontal
	<0.001	0.438	74	-24	14	Right	Superior temporal
	0.003	0.438	58	-22	22	Right	Supramarginal
	0.001	0.438	30	-10	72	Right	Superior frontal
	0.002	0.438	18	14	72	Right	Supplementary motor
	0.001	0.438	74	-40	-10	Right	Middle temporal
	0.001	0.438	60	6	8	Right	Inferior frontal
	0.002	0.438	58	4	22	Right	Precentral
Phonemic	0.001	0.878	48	-48	-46	Right	Cerebellum
COWAT	0.001	0.878	28	12	-42	Right	Middle temporal
	0.001	0.878	30	-8	-48	Right	Fusiform
	0.001	0.878	2	8	42	Right	Middle cingulate
MMSE	0.001	0.994	28	66	10	Right	Superior frontal

FDR-corr, false discovery rate-corrected; K-BNT, Korean version of Boston Naming Test; RCFT, Rey Complex Figure Test; SVLT, Seoul Verbal Learning Test; COWAT, Controlled Oral Word Association Test.

## Discussion

The results of our study showed that patients with aMCI compared to normal subjects had lower performances in both CPT A and CPT B. The SPM correlation analyses revealed that decrease in scores of part B correlated with hypometabolism in bilateral precuneus, middle and posterior cingulate gyri.

To accomplish part A of the test, the subjects should use an egocentric (or body-centered) reference frame to represent the spatial locations of objects surrounding them. Therefore, the part A seemed to evaluate their egocentric orientation. The only difference between part A and part B is the subjects’ rotation in part B just before they replace the cards. To accomplish part B of the test, the subjects should use the information on changes in their body direction. In other words, they should process directional signals of the self, or integrate information on the registered external spatial locations of objects with that on their body direction. Therefore, the part B evaluated their exocentric orientation. As a result, in our study, patients with aMCI were impaired in both egocentric and exocentric orientation. Other previous study also evaluated egocentric orientation and exocentric orientation in MCI [11]. In the study, a four-subtest task was used that required the subjects to locate an invisible goal inside a circular arena. The patients with multiple domain aMCI were impaired on all subtests, while the single domain aMCI group was impaired significantly on tests that focused on exocentric orientation and at the beginning of the real space egocentric subtest [11]. Their deficits in egocentric and exocentric orientation may cause topographical disorientation affecting their activities of daily living. A previous report showed that 17 out of the 41 MCI patients (41.4%) experienced topographical disorientation [12]. Another previous study compared patients with MCI with matched controls on a route learning task, and concluded that while MCI patients recognized landmarks as effectively as controls, they could not find their locations on maps or recall the order in which they were encountered [13].

Our SPM correlational analysis showed that the scores of CPT B correlated with hypometabolism of the posterior cingulate region (p < 0.005). We made two possible explanations for our result. First, it is assumed that the part B of the CPT is the specific test for HD and the posterior cingulate gyrus is the neural correlate of the CPT B. Our results agreed with the results of the case studies which originally mentioned the CPT [3]. In rodents, head direction cells (h cells) that are excited when rats are maintaining a certain heading or orientation within an environment are found in the retrosplenial cortex in addition to several neural structures, such as the anterior dorsal nucleus of the thalamus, lateral dorsal thalamus, lateral mammillary nuclei, striatum, and posterior subiculum [14,15]. These neural substrates may constitute a functional circuit dealing with directional signals of the self [15]. Second, it seems that the CPT B is reflective of levels of general cognitive deficit in patients and hypometabolism in the posterior cingulate gyrus is correlated with levels of their cognition. A previous study compared regional cortical thickness between single domain aMCI and multiple domain aMCI [16]. The study suggested that multiple domain aMCI is a transitional state between single domain aMCI and AD, and that the cortical thinning is evidence that the precuneus is responsible for the multiple cognitive impairments in multiple domain aMCI. Although both possible explanations for our result suggest that the CPT B may reflect the functional status of the posterior cingulate gyrus, we performed further correlational analyses with MMSE and neuropsychological tests covering each cognitive domain and metabolism on FDG-PET to distinguish two hypotheses. We found only correlation between posterior cingulate gyrus and the CPT B as shown in the Table 2 and Table 3. Therefore, our result may be supportive for the first hypothesis which the CPT B is the specific test of heading disorientation dependent on the posterior cingulate gyrus.

We found associations between the CPT B and bilateral posterior cingulate gyrus and precuneus, but the original report from Hashimoto et al. [3] found impairment of the CPT B in patients with only right posterior cingulate gyrus/precuneus lesions indicating that the right retrosplenial region is the critical site for heading disorientation. In addition, several studies reported that damage to the left retrosplenial region is not associated with heading disorientation but with episodic memory deficits [17]. In our study, any memory-related tests did not show correlation with metabolism in the posterior cingulate gyrus. We think that the study needs to be done to evaluate whether the CPT would be also impaired in patients with damage to the left retrosplenial region.

We recognize that there are some limitations to our study. First, we recruited the controls who were free of cognitive problems, which was confirmed by no cognitive complaints as well as by not scoring less than the 16^th^ percentile of the norms for the age-, sex-, and education-matched normal subjects on the Korean version of the Mini-Mental State Examination (MMSE). However, we should admit that we could not exclude the possibilities that the controls might have cognitive impairment which could be detected only by detailed neuropsychological tests. Second, our study recruited 10 (66.7%) single domain aMCI and five (33.3%) multiple domain aMCI patients. Considering the result from the previous MRI morphometric study that the precuneus is responsible for the multiple cognitive impairments in multiple domain aMCI patients, we should have evaluated only patients with multiple domain aMCI patients. Including also patients with single domain amnestic MCI might lead to non-significant results due to type 2 error. Third, because of the limited number of the patients, we used uncorrected p-values for the correlational analysis between the PET images and CPT scores. For this reason, these findings cannot be generalized to the broader community based on this study alone and studies with larger samples are necessary.

## Conclusions

This study suggests that the CPT B may reflect the functional status of the posterior cingulate gyrus in patients with aMCI.

## Competing interests

There are no financial or non-financial competing interests on this study.

## Authors’ contributions

SJL, YSA, TSL, and SYM carried out the study, analyzed the data and produced the draft. All authors read and approved the final manuscript.

## Pre-publication history

The pre-publication history for this paper can be accessed here:

http://www.biomedcentral.com/1471-2377/14/123/prepub
